# Disparities Old and New in US Mental Health during the COVID‐19 Pandemic*

**DOI:** 10.1111/1475-5890.12244

**Published:** 2020-11-30

**Authors:** Zachary Swaziek, Abigail Wozniak

**Affiliations:** ^1^ Federal Reserve Bank of Minneapolis; ^2^ Federal Reserve Bank of Minneapolis; NBER; IZA

**Keywords:** COVID‐19, COVID Impact Survey, depression, disparities, Household Pulse Survey, mental health

## Abstract

The COVID‐19 pandemic has reduced well‐being and economic security on a number of dimensions, likely worsening mental health. In this paper, we assess how mental health in the US population has changed during the pandemic. We use three large, nationally representative survey sources to provide a picture of mental health prior to and during the pandemic. We find dramatic but broad‐based declines in the level of mental health from pre‐pandemic baseline measures across both people and places. Rates of poor mental health have jumped roughly 25 percentage points, from a base of roughly one‐third. We document substantial disparities in mental health but show that the pandemic has generally preserved, rather than widened, these. Significant worsening in relative mental health among Hispanics and respondents aged 30 and older are exceptions. Consistent with an important role for pandemic‐specific shocks, We find that income loss, food insecurity, COVID‐19 infection or death in one's close circle, and personal health symptoms are all associated with substantially worse mental health. If anything, the decline in mental health is worsening as the pandemic wears on and is becoming less related to local COVID‐19 case rates.

## I Introduction

Almost from the beginning of the COVID‐19 pandemic in the United States, observers have expressed concern about the mental health consequences of the pandemic. There are good reasons for this concern. The overall strain of facing a serious disease that is readily transmissible through everyday activities is likely to be significant. Exposure to other rare but unpredictable risks that one can incur in everyday life, such as crime, are known to depress mental health.^1^ The indirect effects of COVID‐19 on well‐being, through recommended mitigation measures and lockdowns, also have the potential to negatively affect mental health. For example, unemployment, which has spiked dramatically, is associated with worsened mental health.^2^

These effects also have the potential to fall unequally on different groups. Service occupations employ many young, less‐educated and minority workers and have been heavily affected by layoffs. At the same time, lower‐income, minority and older individuals are at greater risk from COVID‐19 complications because of their disproportionate rates of suspected risk factors. This may cause greater strain for these groups as they weigh the additional risk in daily life alongside the challenges of keeping themselves safe. Geographic disparities are also likely to emerge. Cities and states differ in many ways that can contribute to COVID‐19 risk as well as the economic and social costs of mitigation, such as for example, segregation, pre‐pandemic inequality, and connectedness between neighbourhoods and cities.

To investigate pandemic‐era changes in mental health in the United States, We draw on evidence from two pandemic‐era survey sources: the Data Foundation's COVID Impact Survey (CIS) and the Census Bureau's Household Pulse Survey (HPS). We measure mental health using an indicator for recent poor mental health experiences that we show is comparable across sources. Both surveys launched in late April 2020 with similar mental health modules, and both ask respondents about the economic and financial changes they have experienced during the pandemic. Beyond that, the surveys offer different advantages and disadvantages for this analysis. The CIS asks detailed questions about current physical symptoms, exposure to COVID‐19 and behavioural changes; however, it is a smaller survey with less regular administration than the HPS. The HPS is much larger and has coverage for 12 consecutive weeks following its introduction, but a number of factors – potentially important for mental health – are only asked in the CIS. We combine our analysis of pandemic‐era sources with a major pre‐pandemic benchmark survey source, the Behavioral Risk Factor Surveillance System (BRFSS).

We first construct a data set that allows us to analyse aggregate local mental health outcomes at the place level, i.e. for state and metropolitan area populations. We explore whether pre‐pandemic place characteristics and local COVID‐19 caseloads are associated with changes in local mental health from the BRFSS baseline. We then turn to regressions using individual‐level data to document disparities across demographic groups in mental health, as well as to assess whether these disparities have changed during the pandemic. Finally, we use the same microdata analysis to explore the relationship between pandemic‐era shocks to financial security, health or behaviour patterns and mental health.

We report several findings. We find dramatic declines in mental health from pre‐pandemic baseline measures across both people and places in both sources. Rates of poor mental health have jumped roughly 25 percentage points, from a base of roughly one‐third. Place‐level variation in poor mental health has increased but is not well explained by local characteristics that precede the pandemic. Local mental health is only modestly related to local COVID‐19 cases and, if anything, this relationship is weakening further as the pandemic progresses.

Analysis by demographic groups also shows large declines in mental health in the COVID‐19 era. Individual‐level analysis identifies several groups with both significantly worse and better reported mental health states than average. However, we show that the pandemic has generally preserved, rather than widened, these mental health disparities. Significant worsening in relative mental health among Hispanics and respondents aged over 30 are exceptions. We also find that several pandemic‐era specific experiences – including income loss, food insecurity, COVID‐19 infection or death in one's close circle, and personal health symptoms – are associated with substantially worse mental health. These experiences are associated with a decline in mental health that is large in magnitude. The impacts of these factors are in many cases roughly comparable to the average 20 percentage point increase in poor mental health. We conclude that economic shocks, health concerns, and personal loss and risk also compound the general decline in mental health for those who experience them, but these are not confined to particular demographic groups.

This paper aligns with papers studying the disparate effects of COVID‐19 on mental health and other outcomes. Several studies document features of mental health in the United States during the pandemic.^3^ Generally, these studies find that US mental health worsened considerably beginning with the onset of the pandemic.^4^ Czeisler et al. (2020) also explore disparities in mental health in the United States using a survey approach and therefore their study is closely related to this paper. However, there are a number of key differences. In this paper, we explore the geographic dimension of mental health declines in addition to individual and subpopulation declines that Czeisler et al. also explore. Also, the data in Czeisler et al. come from an Internet‐based survey, but the sample frame and recruitment procedures were not reported. By contrast, the data we use in this paper come from surveys in which respondents are recruited from address‐based frames or standing survey panels with known representativeness properties. It is possible these sample differences account for some of the qualitative differences in our results.

A number of other papers explore disparities in other pandemic era outcomes. Montenovo et al. (2020) use CPS data to describe disparities in employment declines in the early weeks of the pandemic. Cajner et al. (2020) use proprietary payroll data to study the same outcome. Although our methods are substantively different, like Montenovo et al. (2020) and Lozano Rojas et al. (2020), we also conclude that place characteristics – either pre‐pandemic or pandemic era – are not predictive of the severity of local effects from the switch to the COVID‐19 environment. Alstadsaeter et al. (2020) find similar demographic and socio‐economic disparities in employment effects early in Denmark's COVID‐19 outbreak. Alon et al. (2020) discuss likely disparate effects by gender, using evidence from pre‐pandemic data.

## II COVID Impact Survey and Household Pulse Survey

The HPS and the CIS were both launched in the first month of the pandemic with similar goals. Both are designed to assess a broad spectrum of well‐being measures at high frequency in order to deliver timely data on the pandemic's effects. The two surveys are similar in length, taking about 15–20 minutes to complete, and both make extensive use of questions from other data sources in order to allow comparisons with pre‐pandemic benchmarks.^5^ Both surveys interviewed respondents in weekly waves beginning the week of 20 April 2020. Two subsequent waves of the CIS were administered in the weeks of 4 May and 31 May. Our analysis uses Phase 1 of the HPS, which was administered weekly from 23 April until 21 July.

Despite these many similarities, the two sources differ in their particular scope and scale. The CIS^6^ tracks physical symptoms and COVID‐19 exposure alongside measurement of mitigation behaviours, experience with restrictions to daily life, and a set of well‐being indicators encompassing economic security, and mental and social health. These questions are absent from the HPS, although it does contain more questions about housing security and disruptions to schooling.^7^ The HPS is also much larger in scale than the CIS. Roughly 2,000 CIS respondents per wave come from the AmeriSpeak Panel. These comprise the nationally representative portion of the sample (or the national sample). The CIS also includes roughly 6,500 respondents per wave from address‐based oversamples of 18 subnational areas, which we refer to as ‘places’. These consist of ten states (CA, CO, FL, LA, MN, MO, MT, NY, OR and TX) and eight metropolitan statistical areas (Atlanta, Baltimore, Birmingham, Chicago, Cleveland, Columbus, Phoenix and Pittsburgh). By contrast, the HPS has about 40,000–125,000 unique respondents per weekly wave, and can produce representative statistics for all state populations and 15 large metropolitan areas.^8^

## III Aggregate incidence of poor mental health in the United States, pre‐pandemic and in the pandemic era

The two surveys administer similar mental health modules to respondents. This allows us to construct comparable measures of mental health in both sources. To understand the measurement of mental health across surveys, Table 1 reports aggregate responses on specific mental health components for the surveys used in this paper. This includes mental health items from the 2018 BRFSS as well as the CIS and HPS. Table 1 shows good overlap in question items across all three surveys. Two question items on the CIS are identical to those on the HPS and BRFSS, and all mental health items on the HPS are identical to those in the BRFSS. The survey items all ask about incidence on a five‐point scale, but there are differences in low incidence reporting. HPS and BRFSS allow for ‘zero days’ or ‘several days’ as the lowest incidence categories; CIS allows for ‘Not at all or less than 1 day’ or ‘1–2 days’. Finally, the table shows that the surveys use somewhat different reference frames, with the CIS and HPS using a seven‐day look‐back period and the BRFSS using 30 days for the general mental health item and 14 days for specific mental health issues.

**TABLE 1:** Share reporting positive days of poor mental health, by dimension

*Source*	*CISW1* *(20/4)*	*CISW2* *(4/5)*	*CISW3* *(30/5)*	*HPS W1* *(20/4)*	*HPSW2* *(4/5)*	*HPSW3* *(30/5)*	*BRFSS*
Source year	2020	2020	2020	2020	2020	2020	2018
Reference window	7 days	7 days	7 days	7 days	7 days	7 days	30 (14) days
Over the last X days, have you felt/been bothered by…							
Nervous, anxious, on edge	0.41	0.35	0.40	0.64	0.64	0.64	0.34
Depressed/down, depressed, or hopeless	0.40	0.37	0.40	0.48	0.51	0.52	0.27
Lonely	0.40	0.39	0.40				
Hopeless	0.41	0.38	0.39				
Little pleasure in doing things				0.53	0.54	0.53	0.27
Not able to stop, control worry				0.54	0.54	0.55	0.31
Had physical [anxiety] reactions	0.14	0.09	0.09				
Share of any recent poor mental health days?	0.64	0.61	0.62	0.68	0.67	0.67	0.36

*Note*: W1, W2 and W3 correspond as closely as possible to weeks of CIS administration in 2020. The BRFSS 2018 reference window is the past 14 days for specific mental health conditions and 30 days for any poor mental health days; all other measures use a seven‐day reference window. The BRFSS and HPS use the question construction: ‘how often have you been bothered by…’. The CIS uses: ‘how often have you felt…’. If there are two wording constructions in the first column, the first is the CIS version and the second is the HPS and BRFSS version.

Table 1 reports the shares of survey respondents reporting a positive number of days for each mental health issue during the reference period. Specifically, this is the share of respondents reporting the second‐lowest or higher incidence category during the reference period. The table reports rates for each week of the CIS and for the analogous week (by date) in the HPS. The table shows that rates of mental health issues are strictly greater in both pandemic‐era sources than in the pre‐pandemic BRFSS data for comparable items. These differences are also typically large. Rates of some depression are almost double in the HPS and almost 50 per cent higher in the CIS, compared with 2018. This illustrates another feature of these data: rates of mental health issues are substantially higher in the HPS than in the CIS. Finally, the table shows that rates of extreme distress – measured as a physical anxiety reaction – are lower than those for other mental health states. They also decline across the three focus weeks. However, this item is only available in the CIS.

Although there is considerable overlap in question wording and response options, there are limits to comparability across these surveys. The differences in the reference windows might lead to rates of mental health issues that are lower in the pandemic‐era surveys than the BRFSS. If so, the true 14‐day incidence of mental health issues in the pandemic‐era data might be higher than that reported in the table. Hence, the table may understate pre‐pandemic and pandemic‐era differences in aggregate mental health. It is also unclear whether differences in the low incidence categories across the CIS and HPS/BRFSS contribute to these differences. On the one hand, more respondents are eligible to report positive days of mental health issues when the lowest category is strictly zero, which would lead to lower rates of reporting in the CIS than in the other two sources. On the other hand, respondents with very low incidence may be reluctant to report ‘several’ days of an issue and hence round down to the zero category, leading to lower rates of reporting in the HPS/BRFSS relative to the CIS. Ultimately, we conclude that these sources are sufficiently comparable as to be informative, despite these limitations.

Another limit to comparability is that the specific mental health items in Table 1 are only asked of a small subset of BRFSS respondents from three states/geographic areas. Although the specific mental health items and responses are comparable across the three surveys, the small number of responses to these questions in the BRFSS limits subgroup analysis, and the small number of locations represented limits geographic representativeness. To improve comparability, we create an indicator for any poor mental health days in the reference window that is comparable with another related question in the BRFSS. Specifically, we use responses in the CIS and HPS to classify respondents as having any days of poor mental health in the reference window if they report more than one day of nervousness, anxiety, depression, hopelessness or loneliness. This is equivalent to any days of the CIS's four mental health issues or any days of HPS's first two mental health issues in Table 1. The BRFSS asks a question of all respondents that is closely related to this indicator, which allows us to classify BRFSS respondents as having any poor mental health days in the reference window.^9^ We report the shares of respondents with recent poor mental health by this indicator in each survey in the bottom row of Table 1. The table shows that levels of poor mental health are comparable across the HPS and CIS using this classification. Consistent with specific items being elevated in the COVID‐19 era, this broader indicator is also elevated relative to its level in the pre‐pandemic BRFSS.

**FIGURE 1:**
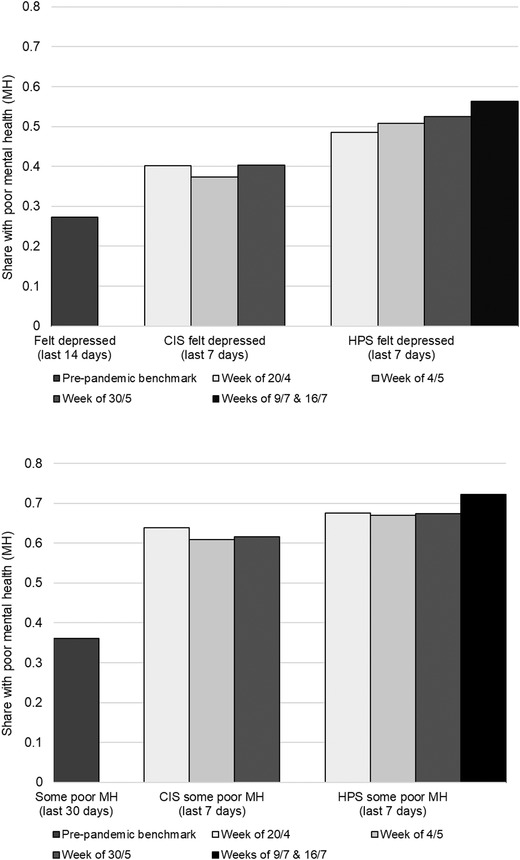
Share reporting depression or any poor mental health status. *Source*: BRFSS (pre‐pandemic benchmark); CIS, all waves; HPS, waves corresponding to CIS survey weeks and average of most recent two weeks.

For ease of comparison, this indicator for any recent poor mental health is the preferred outcome measure that we will use for analysis going forward.^10^ To understand the main incidence differences better across sources, we present results for the items across all three surveys graphically in Figure 1. The top panel shows incidence of any days of depression, which corresponds to the second row of estimates from Table 1. The bottom panel shows incidence of any poor mental health days, using the indicator described above. The figure shows the same elevated rates of mental health issues in the pandemic‐era that are present in Table 1. The figure also adds an additional mid‐July time period available only in the HPS data. These are the most recent data on mental health available in the COVID‐19 era. The figure shows that mental health continues to worsen as the pandemic wears on in the United States.

## IV Local changes in mental health from pre‐pandemic baselines

We begin by examining which places in the CIS sample of states and cities have experienced the biggest changes in mental health from the pre‐pandemic environment. This analysis allows us to assess whether recent mental health changes have been more severe in certain types of places.

**FIGURE 2:**
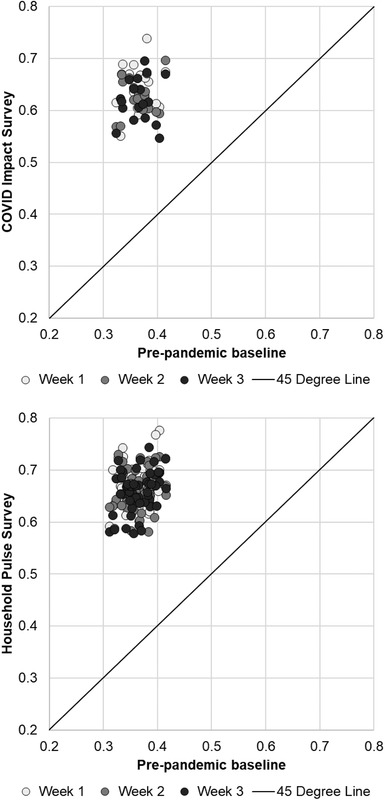
Changes in poor mental health indicators from pre‐pandemic baseline (state and metropolitan area level) *Note*: Baseline levels (BRFSS) indicated on the *x*‐axis. The *y*‐axis indicates pandemic‐era level measured in the CIS (top panel) and HPS (bottom panel). *Source*: CIS, HPS, and BRFSS.

We first compare pre‐pandemic and pandemic‐era place‐level measures of average mental health. Pre‐pandemic means are constructed using the 2018 and 2017 BRFSS.^11^ Figure 2 plots comparison scatterplots using the two pandemic‐era data sources. Each point in the figure represents a place‐level (state or metropolitan area) and week‐level observation on the poor mental health indicator, plotted against the pre‐pandemic baseline for that place. There are several points to take away from this figure. First, mental health has worsened in every place in the HPS and CIS, as implied by the cloud of points above the 45‐degree (equality) line. Second, there is more heterogeneity in pandemic‐era mental health across places than in the baseline measures, as indicated by greater dispersion along the *y*‐axis than the equivalently scaled *x*‐axis. Place‐level dispersion in poor mental health rates has roughly doubled in the COVID‐19 era. Finally, the scatters by week have considerable overlap, indicating little temporal pattern in mental health.

The figures show widespread declines in the level of local mental health, but have certain types of places experienced more substantial changes in these measures than others? To assess this, we run place‐level regressions of the measured changes from baseline (the difference between the *x*‐ and *y*‐axis values for a place from Figure 2) on a range of pre‐pandemic place characteristics. These include simple regional indicators from the HPS and CIS data as well as several variables for pre‐pandemic characteristics measured using external data sources. These include local earnings inequality, median income, segregation between black and white people, geographical connectedness across counties measured using cell phone data, share of employment that is teleworkable, and employment share in the service occupations. Details on sources for these measures can be found in the note to Table 2.

**TABLE 2:** Local mental health and place characteristics

	HPS		CIS	
	*Share with poor mental health*	*Change in share with poor mental health (from 2018/2017)*	*Share with poor mental health*	*Change in share with poor mental health (from 2018/2017)*
*Panel A*				
Mid‐West	–0.048***	–0.046**	–0.015	–0.057
	(0.010)	(0.016)	(0.019)	(0.035)
South	–0.027**	–0.026	–0.040*	–0.068
	(0.009)	(0.016)	(0.018)	(0.037)
West	–0.022*	–0.030	0.017	–0.013
	(0.011)	(0.016)	(0.020)	(0.035)
MSA indicator	0.037***	0.032*	0.011	0.002
	(0.009)	(0.015)	(0.013)	(0.024)
*R* ^2^	0.39	0.21	0.44	0.31
*Panel B*				
80:20 inequality	0.035***	0.024	–0.010	–0.038
	(0.008)	(0.013)	(0.026)	(0.028)
Log median household income	0.070*	0.097	0.060	0.076
	(0.035)	(0.052)	(0.110)	(0.194)
County connectedness	0.000*	0.000*	–0.001	0.000
	(0.000)	(0.000)	(0.000)	(0.000)
Segregation of black	–0.000	–0.001	–0.000	0.000
and white people	(0.000)	(0.000)	(0.001)	(0.002)
Teleworkable share	0.013	0.006	0.617	0.853
	(0.171)	(0.242)	(0.533)	(0.905)
Service occupation share	0.256	0.558	–0.113	1.594*
	(0.250)	(0.284)	(0.516)	(0.640)
*R* ^2^	0.52	0.47	0.54	0.54
Dependent variable mean	0.67	0.30	0.63	0.28

*Note*: Data are place‐level observations constructed from weighted HPS (CIS) microdata and baseline BRFSS data. Each column‐panel is one linear regression containing covariates shown in the panel. Regressions in columns 3 and 4 contain 18 observations for each CIS place; those in column 1 contain 66 for each HPS place, and those in column 2 contain 54 observations where comparable metropolitan areas could easily be constructed in the BRFSS baseline data. Constant terms are unreported. Dependent variables are indicated in the column headings. MSA indicator is a dummy variable for the metropolitan area place units; all other places are states. Robust standard errors are given in parentheses. Regressions are unweighted. ^***^, ^**^ and ^*^ indicate significance at the 1, 5 and 10 per cent levels, respectively.

*Source*: Variable sources for Panel B. Census published tables (inequality, log median household income, and service occupation share); Couture et al. (2020) data set (county connectedness); Frey (2010) analysis of 2005–2009 American Community Survey (segregation of black and white people); Dingel and Neiman (2020) (teleworkable share).

Because there are only 66 (18) places in the HPS (CIS) data, place‐level analysis can only accommodate a limited number of covariates.^12^ To handle this, we divide the covariates into two sets and run place‐based regressions separately on each set. The results should therefore be interpreted with some caution; *R* ^2^ values are reported for each regression and are somewhat large. The dependent variables are shares reporting the poor mental health indicator. These are the same measures shown in the bottom row of Table 1 calculated at the local level for places.^13^

The results are reported in the two panels of Table 2. The first two columns report results for the HPS; the second two for the CIS. The second and fourth columns subtract each place's pre‐pandemic BRFSS value for share reporting any mental health issue from the dependent variable in columns 1 and 3. This represents the change from baseline in local mental health. The first panel shows some regional variation in mental health in the COVID‐19 era. Regions outside the North‐East report better mental health, and metropolitan areas report worse mental health, but the changes from regressions show that these level differences are only weakly related to changes from baseline. The second panel shows that local mental health in the COVID‐19 era is not strongly related to pre‐pandemic place characteristics beyond the region. Relationships, particularly in the changes in specifications, are generally insignificant or only weakly significant.

The analysis so far shows that pre‐existing place characteristics are not associated with changes in local mental health. Thus, while declines in mental health have been substantial across the United States, and while there is more heterogeneity in mental health across places in the pandemic‐era data than the pre‐pandemic data, it does not appear that certain types of places faced systematically larger declines. However, the geographic spread of COVID‐19 has been uneven, and differences in the incidence of COVID‐19 across places may account for varying levels of local mental health.

**FIGURE 3:**
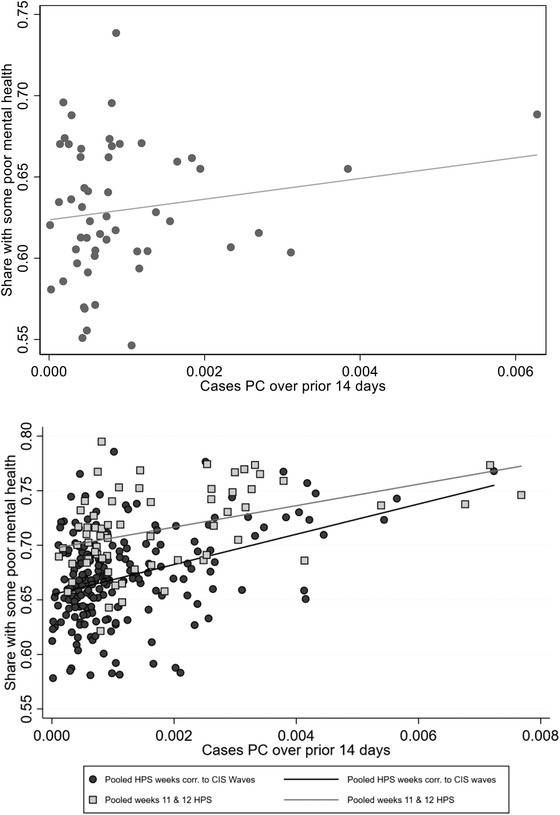
Share of respondents with some poor mental health days and per capita COVID‐19 cases. *Note*: Per capita cases computed as weekly total cases for 14 days prior to beginning of survey week, by place, divided by total population. The top and bottom panels show the CIS and HPS data, respectively. The dark grey and light grey symbols indicate CIS survey weeks and the more recent HPS weeks (late July), respectively. *Source*: CIS and *New York Times* GitHub repository.

We assess this possibility by combining the place aggregates at the weekly level with data on per capita COVID‐19 cases from the *New York Times* GitHub repository of county‐level confirmed cases. In Figure 3, we plot the weekly incidence of some poor mental health against per capita COVID‐19 cases from the preceding two‐week period. The top panel shows a scatterplot using CIS places and poor mental health shares, and the bottom panel uses the HPS. The scatter in the CIS data shows a modest relationship between the two, significant at the 10 per cent level. The bottom panel shows that the relationship between local mental health and local COVID‐19 cases is nearly identical in the HPS during comparable weeks of the pandemic (dark grey circle markers). In the HPS data, we can also examine the final weeks of Phase 1 HPS data, which cover the second half of July (light grey square markers). These show that the relationship between local cases and mental health has, if anything, weakened as the pandemic has progressed in the United States. We conclude that local mental health is only modestly related to local COVID‐19 incidence, and that this relationship is moderating over time. These results align – broadly – with other analyses showing that local policies are poor predictors of local economic conditions in the pandemic period.^14^

## V Factors in individual‐level mental health in the COVID‐19 era

Given concerns about inequitable effects of COVID‐19 on health and economic security for lower‐earning workers and workers of colour, it is important to analyse people in addition to places. Another way to study the effects of COVID‐19 on mental health is to look at the same changes from baseline by demographic group as we examined in the place‐level analysis. Thus, we turn to individual‐level data to assess the role that individual experiences during the pandemic play in determining mental health status.

**FIGURE 4:**
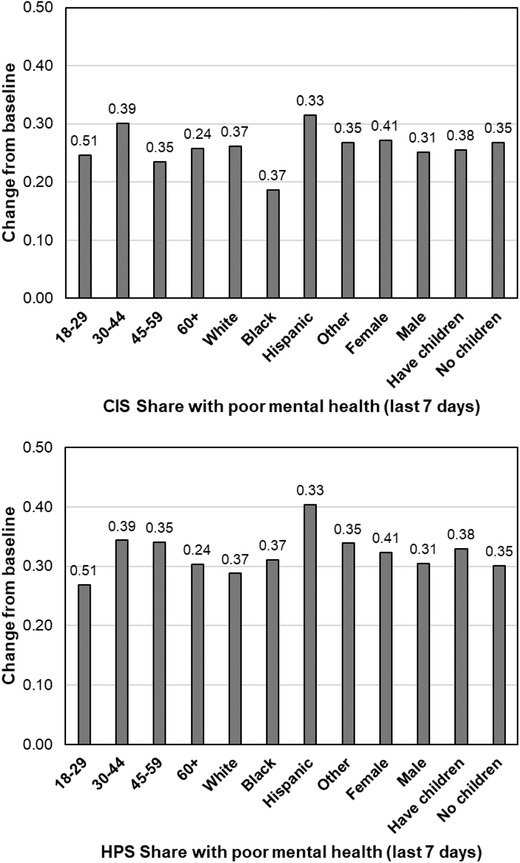
Changes in mental health from pre‐pandemic baseline for select demographic groups. *Note*: Baseline levels are given at the top of each bar. The *y*‐axis indicates change from the pre‐pandemic baseline measured in the CIS (top panel) or HPS (bottom panel). White, Black and Other are all non‐Hispanic. Hispanic includes all Hispanic respondents of any race. All changes are statistically significant at the 1 per cent level. *Source*: CIS, HPS and BRFSS.

For this analysis, we use the national sample component of the CIS and all microdata from the HPS.^15^ We repeat the construction of changes from baseline used in Figure 1 for several demographic groups. The changes from the pre‐pandemic external benchmarks for these subpopulations are shown in Figure 4, again for both the CIS and HPS. The figure shows that declines in mental health are generally widespread across demographic groups, with almost all demographic groups experiencing an increase in rates of poor mental health of more than 20 percentage points. As was the case with the comparisons in Table 1, HPS rates of poor mental health are typically higher than in the CIS.

The group‐level changes in mental health appear very broad, with many groups experiencing large declines from baseline. However, which of these pandemic‐era disparities in mental health persist after controlling for respondent characteristics simultaneously? Also, are any of these disparities more substantial than in normal times?

We begin by assessing disparities in mental health using microdata that allow us to control for multiple respondent characteristics simultaneously. We regress an indicator for experiencing any of the four poor mental health states on a range of demographic and socio‐economic characteristics. The results are reported in Table 3, and in Tables A2–A4 in the online Appendix. Estimates from the HPS, the CIS and the pre‐pandemic BRFSS are reported, as well as estimates from the subsample of labour force participants in each survey. Results are very similar between the full sample and labour force participants in all three surveys.

**TABLE 3:** Poor mental health incidence and demographic and SES characteristics

	*CIS*	*HPS*	*BRFSS*
Black (NH)	–0.075**	–0.040***	–0.055***
	(0.028)	(0.008)	(0.006)
Hispanic	–0.023	0.000	–0.117***
	(0.026)	(0.008)	(0.007)
Other (NH)	–0.039	–0.018*	–0.057***
	(0.034)	(0.007)	(0.007)
Female	0.122***	0.118***	0.108***
	(0.017)	(0.004)	(0.003)
Aged 30–44	–0.061*	–0.020*	–0.097***
	(0.026)	(0.008)	(0.006)
Aged 45–59	–0.178***	–0.074***	–0.163***
	(0.027)	(0.008)	(0.006)
Aged 60+	–0.270***	–0.219***	–0.301***
	(0.026)	(0.007)	(0.005)
No high school diploma	0.020	0.021	0.023**
	(0.039)	(0.013)	(0.007)
Some college education	0.059*	0.031***	0.042***
	(0.024)	(0.006)	(0.005)
College degree or above	0.082***	0.028***	0.028***
	(0.023)	(0.006)	(0.004)
Income $40k–75k	–0.030	–0.055***	–0.075***
	(0.021)	(0.006)	(0.005)
Income $75k+	–0.032	–0.110***	–0.128***
	(0.022)	(0.006)	(0.005)
Two adult only household	–0.013	–0.002	
	(0.022)	(0.007)	
Children, none school age	0.014	–0.010	
	(0.040)	(0.010)	–0.032***
Children, some school age	–0.055*	0.010	(0.004)
	(0.025)	(0.007)	
Other household type	0.027	0.039***	
	(0.026)	(0.008)	
Rural county	0.028		
	(0.033)		
Urban/top 15 MSA	0.027	0.047***	0.033***
	(0.022)	(0.005)	(0.004)
Mid‐West	–0.042	–0.036***	–0.005
	(0.026)	(0.007)	(0.005)
South	–0.088***	–0.040***	–0.014**
	(0.025)	(0.006)	(0.005)
West	–0.055*	–0.013	0.016**
	(0.026)	(0.007)	(0.005)
Dependent variable mean	0.62	0.67	0.36
*N*	6,399	377,550	346,799

*Note*: Data are CIS and HPS microdata; all waves for CIS and weekly waves for HPS corresponding to CIS (1, 2, 3, 5 and 6). Dependent variable in all columns is indicator for positive days with poor mental health in the last week (CIS, HPS) or last 30 days (BRFSS). Columns report coefficients from linear regression. ‘NH’ means not of Hispanic, Latino, or Spanish origin. All analyses are weighted and all variables are categorical. Omitted categories: white non‐Hispanic; male; high school graduate; income under $40,000 per year; suburban county/not residing in top 15 MSA; one adult only household; and North‐East region. ^***^, ^**^ and ^*^ indicate significance at the 1, 5 and 10 per cent levels, respectively.

The estimates in the first two columns of Table 3 show a number of systematic differences in pandemic‐era mental health. First, several groups report significantly worse mental health than others; because the dependent variable is an indicator for poor mental health, these are groups with positive coefficients. Women in the HPS and the CIS report poor mental health at rates much greater than men. The rates of poor mental health for women are 11–12 percentage points, or about 17 per cent, greater than those for men. Younger respondents (those aged 18–29) also report significantly worse mental health than older respondents. This is consistent with elevated levels of suicide ideation reported among young adults in Czeisler et al. (2020).^16^ Poor mental health incidence is also higher among those with some college education or above. Across both surveys, groups who are less likely to report poor mental health include black respondents, older respondents, and those from the South. In the CIS, those with those with school‐aged children are (weakly) less likely to report poor mental health; in the HPS, medium and high earners are much less likely to report poor mental health. The magnitude of the significant differences is also generally large, compared with the mean outcome values at the bottom of the table.

Some of these results are surprising at first. Results indicating lower rates of poor mental health among black respondents are particularly striking, as the disease has disproportionately affected this community. This is possibly due to a level effect in symptom reporting, and is consistent with findings across pre‐pandemic surveys in the epidemiology of mental health.^17^ The negative coefficient on households with school‐aged children in the CIS is also notable, indicating better mental health in these households in this source. Respondents from the South and West also report lower rates of poor mental health.

These patterns are robust across sources in the COVID‐19 era, but do they represent a departure from pre‐pandemic mental health disparities? A partial answer to this is provided in the final column of Table 3, where we repeat the regression analysis using microdata from the benchmark BRFSS. The BRFSS comparison reveals that a number of disparities identified in pandemic‐era reports of mental health are also present in normal times. To assess this more formally, we pool microdata from the BRFSS and pandemic‐era sources and estimate regressions in which the HPS (CIS) regressions of Table 3 are nested in the BRFSS regression. Specifically, the nested regression includes all covariates listed in Table 3, plus a full set of those covariates interacted with an indicator for the pandemic‐era source (either HPS or CIS).^18^

**FIGURE 5:**
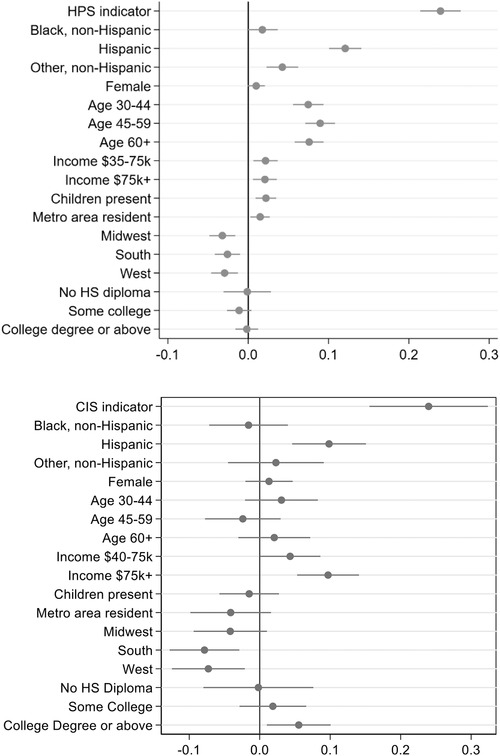
Pandemic‐era changes in relative mental health. *Note*: Coefficients and robust standard errors from a regression of poor mental health status on demographics with nested pre‐pandemic and pandemic‐era coefficients. The panels report the pandemic‐era source level (intercept) and the full set of interactions of demographics with the pandemic‐era data indicator. The top panel reports interactions using HPS data; indicator is labelled HPS, for Household Pulse Survey. The bottom panel uses CIS data. Baseline period data are from BRFSS 2018 microdata, in both panels.

Figure 5 plots coefficients on the pandemic‐era interactions from this nested approach as well as the main effect of the pandemic‐era source (either HPS or CIS). The interactions show how the pandemic‐era coefficients differ from their pre‐pandemic (BRFSS) analogues. Broadly, the HPS and CIS provide a similar picture of which groups have experienced changes in relative mental health in the COVID‐19 era. The large, general worsening in mental health is clear in the large positive coefficients on the main effect of the pandemic‐era for both surveys. The top coefficient in each panel is near 25 percentage points, similar to the raw differences in rates across surveys in Table 1. In both surveys, Hispanic respondents and higher earners have experienced declines in mental health that worsened their mental health relative to other race and ethnic groups or lower earners. Mental health has also worsened for residents in the North‐East relative to residents of other regions, although evidence in Figure 3 from the latest waves of the HPS suggests that this might be moderating.

Consistent with its larger sample size, the HPS coefficients have smaller standard errors and are more often significantly different from their pre‐pandemic values. While the results across the two surveys are similar, the results for age groups are substantively different. HPS data show that mental health has worsened significantly for older respondents relative to the youngest group, though Table 3 shows that younger respondents still have the highest levels of poor mental health across age groups. CIS data show no relative changes in mental health across age groups. The relative worsening of mental health for higher earners is also more muted, though still significant, in the HPS.

Table 3 and Figure 5 establish that poor mental health during the pandemic is more likely among some groups than others, although many of these differences likely reflect underlying mental health disparities that existed prior to COVID‐19. We next turn to questions about how aspects of pandemic life relate to mental health status. In Table 4, we add measures of income loss and current food insecurity during the pandemic to the regressions in Table 3. Coefficients for these new measures are reported in the table; unreported estimates on the demographic and socio‐economic status (SES) controls are similar to those in Table 3. Again, estimates are provided using both HPS and CIS data.

**TABLE 4:** Poor mental health incidence and economic, health vulnerability

	*HPS*	*CIS*	*CIS*	*CIS*
Income loss since March 2020	0.123***	0.045*	0.029	0.030
	(0.005)	(0.020)	(0.020)	(0.020)
Food insecurity	0.182***	0.112***	0.096***	0.085***
	(0.006)	(0.024)	(0.025)	(0.025)
COVID‐19 diagnosis in home			0.154***	0.121**
			(0.039)	(0.039)
Friend/family COVID‐19 death			0.077*	0.073*
			(0.036)	(0.036)
2+ risk factors for severe COVID‐19			0.041*	0.019
			(0.020)	(0.020)
Total protective steps			0.023***	0.022***
			(0.003)	(0.003)
Total restrictions experienced			0.004	0.004*
			(0.002)	(0.002)
2+ COVID‐19 symptoms in last seven days				0.145***
				(0.019)
Demographic, SES controls	Yes	Yes	Yes	Yes
				
*N*	376,540	5,865	5,610	5,609

*Note*: Data are CIS and HPS microdata; all waves for CIS and weekly waves for HPS corresponding to CIS (1, 2, 3, 5 and 6). Dependent variable in all columns is indicator for positive days with poor mental health in the last week. Columns report coefficients from linear regression. Income loss in CIS constructed from hours of work measures and reasons for not working during the past seven days. All analyses are weighted and all variables are categorical. Demographic and SES controls from Table 3 included in all regressions. ^***^, ^**^ and ^*^ indicate significance at the 1, 5 and 10 per cent levels, respectively.

The first two columns of Table 4 show that income loss and food insecurity during the pandemic are associated with a significantly and substantively higher incidence of poor mental health. Income loss and (to a lesser extent) food insecurity during the pandemic are measured differently in the HPS and CIS, as noted in the table notes. However, the results are roughly comparable in magnitude across surveys. The HPS estimates suggest that experiencing food insecurity increases rates of poor mental health to a degree (18 percentage points) that almost equals the average change in mental health from pre‐pandemic to the pandemic era (25 percentage points).

The final two columns of Table 4 examine the role that behavioural responses, underlying risk factors, and personal experiences with COVID‐19 have on mental health. Here, the CIS is the only available source, but the consistency of other estimates across the two survey sources makes it likely that CIS‐only estimates generalise to larger samples. These estimates show that personal experience with a COVID‐19 infection or death both contribute meaningfully to elevated rates of poor mental health.^19^ Individuals with a COVID‐19 diagnosis in their home are about 12–15 percentage points more likely to report poor mental health; this is large compared with the mean rate of 0.62. Having a close friend or family member who has died from COVID‐19 also elevates poor mental health, by about 7 percentage points. Risk factors for severe COVID‐19 also contribute (weakly).

This specification also includes measures of personal behavioural changes during the pandemic, measured as total protective steps taken out of 19 possible steps, as well as personal restrictions experienced, again of our total of 19 possible restrictions. Protective steps are significantly associated with worsened mental health, but the magnitude is modest compared with other items. Experienced restrictions seem to have little relationship to mental health.

The last column in Table 4 adds a control for experiencing COVID‐19 symptoms in the last seven days. These are associated with a large increase in the incidence of poor mental health. Someone experiencing two or more COVID‐19 symptoms is 15 percentage points more likely to report poor mental health, suggesting a substantial mental health tool of these symptoms. It is worth noting that these symptoms are reported at high rates; Ruffini, Sojourner and Wozniak (2020) show that 26 per cent of the population report such symptoms over the past seven days in the CIS.

## VI Conclusion

We use two survey sources, the CIS and the HPS, to study the incidence of poor mental health in the United States during the COVID‐19 era and to compare it to pre‐pandemic baselines.

We report several findings. First, mental health has worsened substantially in the United States since the onset of the pandemic. The rate of poor mental health days has nearly doubled for the US population, rising by 25 percentage points from a base level of roughly one‐third. The decline in mental health is apparent across a broad set of places and individuals. All states have experienced declines in mental health in their populations of roughly this magnitude. We find that place‐level dispersion in mental health has doubled in the pandemic era compared with pre‐pandemic data, but place‐specific factors pre‐dating the pandemic do little to explain this dispersion. We also find that local COVID‐19 infection rates are only modestly related to local mental health levels and, if anything, this relationship has weakened recently.

Analysis of individual‐level data reveals that mental health has worsened for individuals across the board. All identifiable demographic groups report worse mental health in the pandemic era than in a comparable pre‐pandemic source. Moreover, although disparities in mental health were significant in the pre‐pandemic era, the pandemic has generally preserved these rather than widened them. Two substantive exceptions to this are Hispanic respondents and those over 30. Both groups have seen their mental health worsen relative to other groups, compared with their pre‐pandemic levels. Notably, in both cases, this relative decline reflects a narrowing disparity in which mental health worsened in one group to reduce the gap with others. The fact that relative mental health has not worsened for some groups known to experience disproportionate changes to employment and daily life – notably black respondents and women – is something of a surprise. However, further analysis reveals that income loss and food insecurity are associated with large declines in mental health, as is exposure to a COVID‐19 infection, death, or personal COVID‐19 symptoms. Taken together, these results suggest that pandemic‐era shocks to financial security and health meaningfully worsen mental health, but the large, population‐wide declines in mental health swamp most relative changes in mental health across groups.

## Supporting information

• Online Appendix

## Footnotes

1 Dustmann and Fasani, 2016.

2 Krueger and Mueller, 2012; Cziesler et al., 2020.

3 Cziesler et al., 2020; Holingue et al., 2020a, b.

4 Holingue et al., 2020a, b.

5 The two surveys use different sampling frames but, conditional on being contacted to participate, response rates are broadly similar across the two. For more detail on the CIS, see Wozniak (2020) and Benz (2020). For more detail on the HPS, see Fields et al. (2020) and US Census Bureau (2020a,b).

6 Wozniak et al., 2020.

7 Fields et al., 2020.

8 The HPS also includes a longitudinal subsample of respondents repeating the survey in subsequent weeks. This group averages 20 per cent of the overall sample in weeks 2–12. The Census Bureau does not recommend dropping repeat responders (personal communication), so we treat the HPS data as a repeated cross‐section in all analyses. However, the omission of repeat responders does not materially affect the main results in this paper.

9 The BRFSS question is: ‘Now thinking about your mental health, which includes stress, depression, and problems with emotions, for how many days during the past 30 days was your mental health not good?’ Cronin, Forsstrom and Papageorge (2020) show that self‐reporting of general mental health (in their data, from a five‐point scale) are strongly related to reports of the specific poor mental health issues that respondents report in the CIS and HPS. Broadly, the evidence of Cronin et al. is consistent with the common indicator approach we have taken to making pre‐pandemic and pandemic‐era sources comparable.

10 To alleviate any remaining concerns about comparability across the HPS and CIS, in the online Appendix we repeat most analysis using indicators specifically for either depression or anxiety, the two items that are directly comparable across the two pandemic‐era surveys.

11 The 2017 SMART BRFSS MMSA microdata are the most recent with metropolitan area identifiers for substate estimates. The 2017 data are used for constructing pre‐pandemic baselines at the metropolitan area level. The 2018 data are used for constructing baselines at the state level.

12 Given limited inter‐week variation in the dependent variable (and no such variation in the covariates, by construction), we estimate this as a place‐level‐only regression rather than incorporating the weekly time dimension of the HPS and CIS data.

13 Table A1 in the online Appendix repeats the analysis using shares reporting specific mental health issues of anxiety and depression.

14 Alstadsaeter et al., 2020; Montenovo et al., 2020; Han et al., 2020.

15 Weighted analysis of responses from the 18 subnational oversamples in the CIS is also an option, but this cannot produce nationally representative statistics.

16 These patterns are also broadly similar regardless of the mental health indicator used. Tables A1–A3 report estimates from the same regression specifications, using specific mental health issues as the dependent variable.

17 See Barnes, Keyes and Bates (2013) for a partial review.

18 Robust standard errors are reported. The Census Bureau recommends using replicate weights to calculate standard errors in the HPS, but replicate weights are not provided for either the CIS or BRFSS.

19 It is worth noting that small shares of CIS respondents report these two experiences. Nearly 5 per cent of CIS respondents report that a close friend or family member has died of COVID‐19 or recent respiratory illness, and 1.2 per cent report a COVID‐19 infection in their home. COVID‐19 symptoms (analysed in the final column of Table 4) are reported by many more respondents: 26 per cent report experiencing two or more COVID‐19 symptoms in the last seven days. Ruffini et al. (2020) examine self‐reported symptoms during the pandemic in detail.
