# Supplementary material for: Disparities Old and New in US Mental Health during the COVID‐19 Pandemic
Source: Fisc Stud. 2020 Nov 30;41(3):709–32. doi: 10.1111/1475-5890.12244 (PMC7753757; doi:10.1111/1475-5890.12244)
Supplement: Supplementary file 1 — • Online Appendix [file FISC-41-709-s001.docx]

Disparities Old and New in US Mental Health during the COVID-19 Pandemic: Online Appendix

Zachary Swaziek† and Abigail Wozniak‡

† *Federal Reserve Bank of Minneapolis*

*(*[*zachswaz@gmail.com*](mailto:zachswaz@gmail.com)*)*

‡ *Federal Reserve Bank of Minneapolis; NBER; IZA*

*(abigailwozniak@gmail.com)*

*TABLE A1*

*Local mental health and place characteristics, specific mental health items*

|  | HPS | | CIS | |
| --- | --- | --- | --- | --- |
|  | *Share with*  *depression* | *Share with*  *anxiety* | *Share with*  *depression* | *Share with*  *anxiety* |
| *Panel A* |  |  |  |  |
| Mid-West | –0.027* | –0.050*** | –0.033 | –0.006 |
|  | (0.012) | (0.011) | (0.016) | (0.022) |
| South | –0.002 | –0.034*** | –0.044* | –0.033 |
|  | (0.011) | (0.008) | (0.019) | (0.020) |
| West | –0.012 | –0.025* | 0.003 | 0.003 |
|  | (0.012) | (0.010) | (0.018) | (0.022) |
| MSA indicator | 0.027** | 0.036*** | 0.010 | 0.004 |
|  | (0.009) | (0.009) | (0.012) | (0.013) |
| *R*^2^ | 0.22 | 0.40 | 0.47 | 0.30 |
| *Panel B* |  |  |  |  |
| 80:20 inequality | 0.035*** | 0.032*** | 0.017 | 0.012 |
|  | (0.008) | (0.009) | (0.026) | (0.023) |
| Log median household income | 0.008 | 0.071 | –0.031 | 0.083 |
|  | (0.033) | (0.038) | (0.117) | (0.118) |
| County connectedness | 0.000 | 0.000 | –0.001 | –0.000 |
|  | (0.000) | (0.000) | (0.001) | (0.000) |
| Segregation of black | 0.000 | –0.000 | –0.001 | –0.000 |
| and white people | (0.000) | (0.000) | (0.001) | (0.001) |
| Teleworkable share | –0.006 | –0.001 | 0.866 | 0.322 |
|  | (0.143) | (0.197) | (0.555) | (0.578) |
| Service occupation share | 0.102 | 0.216 | –0.302 | –0.401 |
|  | (0.234) | (0.268) | (0.758) | (0.454) |
| *R*^2^ | 0.42 | 0.45 | 0.37 | 0.47 |
|  |  |  |  |  |
| Dependent variable mean | 0.50 | 0.63 | 0.39 | 0.39 |

*Note*: Same as for Table 2.

*TABLE A2*

*Incidence of specific mental health issues and demographic and SES characteristics: CIS*

|  | *Depression* | *Anxiety* | *Depression*  *LF only* | *Anxiety,*  *LF only* |
| --- | --- | --- | --- | --- |
| Black (NH) | –0.065* | –0.085** | –0.029 | –0.064 |
|  | (0.027) | (0.026) | (0.035) | (0.033) |
| Hispanic | 0.003 | –0.005 | –0.006 | –0.001 |
|  | (0.027) | (0.026) | (0.033) | (0.031) |
| Other (NH) | –0.008 | –0.056 | 0.006 | –0.051 |
|  | (0.034) | (0.035) | (0.042) | (0.043) |
| Female | 0.087*** | 0.092*** | 0.077*** | 0.089*** |
|  | (0.017) | (0.017) | (0.022) | (0.021) |
| Aged 30–44 | –0.034 | –0.058* | –0.031 | –0.068* |
|  | (0.030) | (0.029) | (0.032) | (0.031) |
| Aged 45–59 | –0.123*** | –0.180*** | –0.119*** | –0.181*** |
|  | (0.030) | (0.029) | (0.034) | (0.033) |
| Aged 60+ | –0.249*** | –0.283*** | –0.278*** | –0.306*** |
|  | (0.028) | (0.028) | (0.035) | (0.036) |
| No high school diploma | 0.064 | 0.041 | 0.059 | 0.072 |
|  | (0.041) | (0.041) | (0.056) | (0.054) |
| Some college education | 0.062** | 0.045 | 0.066* | 0.085** |
|  | (0.024) | (0.024) | (0.031) | (0.030) |
| College degree | 0.042 | 0.036 | 0.035 | 0.066* |
| or above | (0.023) | (0.023) | (0.030) | (0.028) |
| Income $40k–75k | –0.015 | –0.024 | –0.033 | –0.046 |
|  | (0.022) | (0.022) | (0.029) | (0.028) |
| Income $75k+ | –0.029 | –0.057* | –0.029 | –0.067* |
|  | (0.023) | (0.023) | (0.030) | (0.028) |
| Two adult only | –0.034 | –0.001 | –0.037 | 0.006 |
| household | (0.022) | (0.021) | (0.029) | (0.029) |
| Children, | –0.028 | –0.041 | –0.037 | –0.040 |
| none school age | (0.045) | (0.043) | (0.050) | (0.048) |
| Children, | –0.045 | –0.018 | –0.041 | –0.018 |
| some school age | (0.026) | (0.026) | (0.030) | (0.029) |
| Other household type | 0.014 | 0.031 | 0.025 | 0.054 |
|  | (0.028) | (0.027) | (0.035) | (0.033) |
| Rural county | –0.001 | 0.032 | –0.044 | 0.024 |
|  | (0.033) | (0.033) | (0.041) | (0.042) |
| Urban/top 15 MSA | 0.016 | 0.006 | 0.007 | 0.013 |
|  | (0.022) | (0.022) | (0.028) | (0.027) |
| Mid-West | –0.080** | –0.083** | –0.095** | –0.132*** |
|  | (0.028) | (0.028) | (0.034) | (0.033) |
| South | –0.090*** | –0.128*** | –0.080* | –0.164*** |
|  | (0.027) | (0.026) | (0.033) | (0.032) |
| West | –0.084** | –0.076** | –0.074* | –0.104** |
|  | (0.028) | (0.027) | (0.035) | (0.033) |
| Dependent variable mean | 0.39 | 0.39 | 0.42 | 0.42 |
| *N* | 6,404 | 6,410 | 4,332 | 4,339 |

*Note*: Data are CIS microdata, all waves. Dependent variable in all columns is indicator for positive days of the indicated poor mental health experience (depression or anxiety) in the last week. Columns report coefficients from linear regression. All analyses are weighted and all variables are categorical. Omitted categories are: white non-Hispanic; male; high school graduate; income under $40,000 per year; suburban county; one adult only household; and North-East region. NH indicates non-Hispanic. ***, ** and * indicate significance at the 1, 5 and 10 per cent levels, respectively.

*TABLE A3*

*Incidence of specific mental health issues and demographic and SES characteristics: HPS*

|  | *Depression* | *Anxiety* | *Depression*  *LF only* | *Anxiety,*  *LF only* |
| --- | --- | --- | --- | --- |
| Black (NH) | –0.037*** | –0.052*** | –0.046*** | –0.057*** |
|  | (0.008) | (0.008) | (0.009) | (0.009) |
| Hispanic | –0.010 | 0.000 | –0.024* | –0.008 |
|  | (0.008) | (0.008) | (0.009) | (0.009) |
| Other (NH) | 0.007 | –0.025*** | 0.006 | –0.027*** |
|  | (0.008) | (0.007) | (0.009) | (0.008) |
| Female | 0.082*** | 0.126*** | 0.084*** | 0.129*** |
|  | (0.005) | (0.004) | (0.005) | (0.005) |
| Aged 30–44 | –0.024** | –0.015 | –0.023* | –0.015 |
|  | (0.009) | (0.008) | (0.009) | (0.009) |
| Aged 45–59 | –0.073*** | –0.063*** | –0.075*** | –0.063*** |
|  | (0.009) | (0.008) | (0.009) | (0.008) |
| Aged 60+ | –0.207*** | –0.218*** | –0.168*** | –0.157*** |
|  | (0.008) | (0.008) | (0.010) | (0.009) |
| No high school diploma | 0.030* | 0.006 | 0.028 | –0.003 |
|  | (0.014) | (0.014) | (0.016) | (0.016) |
| Some college education | 0.014* | 0.037*** | 0.014 | 0.038*** |
|  | (0.007) | (0.006) | (0.008) | (0.008) |
| College degree or above | –0.014* | 0.033*** | –0.020** | 0.031*** |
|  | (0.006) | (0.006) | (0.007) | (0.007) |
| Income $35k–75k | –0.083*** | –0.048*** | –0.084*** | –0.046*** |
|  | (0.007) | (0.006) | (0.008) | (0.008) |
| Income $75k+ | –0.166*** | –0.102*** | –0.174*** | –0.104*** |
|  | (0.007) | (0.006) | (0.008) | (0.007) |
| Two adult only household | –0.016* | 0.005 | –0.016* | 0.009 |
|  | (0.007) | (0.007) | (0.008) | (0.008) |
| Children, none school age | –0.059*** | 0.002 | –0.064*** | 0.003 |
|  | (0.010) | (0.010) | (0.012) | (0.011) |
| Children, some school age | –0.026*** | 0.021** | –0.034*** | 0.014 |
|  | (0.008) | (0.007) | (0.009) | (0.008) |
| Other household type | 0.021** | 0.044*** | 0.018* | 0.044*** |
|  | (0.008) | (0.008) | (0.009) | (0.009) |
| Top 15 MSA | 0.039*** | 0.045*** | 0.040*** | 0.043*** |
|  | (0.005) | (0.005) | (0.006) | (0.006) |
| Mid-West | –0.028*** | –0.034*** | –0.024** | –0.027*** |
|  | (0.007) | (0.007) | (0.008) | (0.008) |
| South | –0.027*** | –0.039*** | –0.025** | –0.034*** |
|  | (0.007) | (0.007) | (0.008) | (0.007) |
| West | –0.011 | –0.009 | –0.005 | –0.001 |
|  | (0.007) | (0.007) | (0.008) | (0.008) |
| Dependent variable mean | 0.51 | 0.64 | 0.53 | 0.67 |
| *N* | 378,053 | 377,892 | 286,541 | 286,475 |

*Note*: Data are HPS microdata, weeks 1, 2, 3, 5 and 6. Dependent variable in all columns is indicator for positive days of the indicated poor mental health experience (depression or anxiety) in the last week. Columns report coefficients from linear regression. All analyses are weighted and all variables are categorical. Omitted categories are: white non-Hispanic; male; high school graduate; income under $35,000 per year; not residing in a top 15 MSA; one adult only household; and North-East region. NH indicates non-Hispanic. ***, ** and * indicate significance at the 1, 5 and 10 per cent levels, respectively.

*TABLE A4*

*Poor mental health incidence and demographic and SES characteristics, labour force (LF) only subsamples*

|  | *CIS, LF only* | | *HPS, LF only* | | *BRFSS, LF only* | | |
| --- | --- | --- | --- | --- | --- | --- | --- |
| Black (NH) | –0.068* | (0.034) | –0.045*** | (0.009) | 0.075*** | (0.008) |  |
| Hispanic | –0.035 | (0.030) | –0.006 | (0.009) | 0.104*** | (0.008) |  |
| Other (NH) | –0.036 | (0.040) | –0.018* | (0.008) | 0.079*** | (0.009) |  |
| Female | 0.111*** | (0.020) | 0.119*** | (0.005) | 0.122*** | (0.004) |  |
| Aged 30–44 | –0.059* | (0.028) | –0.020* | (0.008) | 0.101*** | (0.007) |  |
| Aged 45–59 | 0.176*** | (0.030) | –0.074*** | (0.008) | 0.191*** | (0.007) |  |
| Aged 60+ | 0.268*** | (0.036) | –0.166*** | (0.009) | 0.292*** | (0.007) |  |
| No high school diploma | 0.044 | (0.050) | 0.016 | (0.015) | 0.006 | (0.011) |  |
| Some college education | 0.078* | (0.030) | 0.033*** | (0.007) | 0.045*** | (0.006) |  |
| College degree or above | 0.087*** | (0.028) | 0.027*** | (0.007) | 0.046*** | (0.006) |  |
| Income $40k–75k | –0.049 | (0.027) | –0.056*** | (0.007) | 0.041*** | (0.007) |  |
| Income $75k+ | –0.036 | (0.028) | –0.113*** | (0.007) | 0.093*** | (0.006) |  |
| Two adult only household | –0.015 | (0.028) | 0.005 | (0.008) |  |  |  |
| Children, none school age | –0.001 | (0.045) | –0.008 | (0.011) | 0.020*** (0.005) | |  |
| Children, some school age | –0.057 | (0.029) | 0.004 | (0.008) |  |  |  |
| Other household type | 0.047 | (0.031) | 0.038*** | (0.009) |  |  |  |
| Rural county | –0.026 | (0.042) |  |  |  |  |  |
| Urban/top 15 MSA | 0.030 | (0.028) | 0.046*** | (0.005) | 0.050*** | (0.005) |  |
| Mid-West | –0.054 | (0.031) | –0.031*** | (0.007) | –0.005 | (0.006) |  |
| South | –0.095** | (0.029) | –0.035*** | (0.007) | –0.020** | (0.006) |  |
| West | –0.075* | (0.030) | –0.007 | (0.007) | 0.025*** | (0.007) |  |
|  |  | |  | |  | |  |
| Dependent variable mean | 0.65 | | 0.71 | | 0.35 | |  |
| *N* | 4,333 | | 286,239 | | 346,799 | |  |

*Note*: Data are CIS and HPS microdata; all waves for CIS and weekly waves for HPS corresponding to CIS. Dependent variable in all columns is indicator for positive days of any of four poor mental health experiences in the last week. Columns report coefficients from linear regression. ‘LF’ indicates the estimating sample is restricted to labour force participants. All analyses are weighted and all variables are categorical. Omitted categories are: white non–Hispanic; male; high school graduate; income under $40,000 per year; suburban county/not residing in top 15 MSA; one adult only household; and North-East region. NH indicates non-Hispanic. ***, ** and * indicate significance at the 1, 5 and 10 per cent levels, respectively.
